# Hepatic Hydrothorax without Any Evidence of Ascites

**DOI:** 10.1100/tsw.2011.68

**Published:** 2011-03-07

**Authors:** Vikram Doraiswamy, Sandeep Riar, Pranabh Shrestha, Justin Pi, Mohammad Alsumrain, Arianne Bennet-Venner, Jennifer Kam, Alan Klukowicz, Richard Miller

**Affiliations:** Department of Pulmonary Medicine, Saint Michael's Medical Center in association with Seton Hall University-School of Health and Medical Sciences, Newark, NJ, USA

**Keywords:** hepatic, hydrothorax, ascites, pleural effusion

## Abstract

Hepatic hydrothorax usually presents in association with ascites, but there are rare cases when it does not. This case helps to support the differential of hepatic hydrothorax in patients who have a history of liver cirrhosis, portal hypertension, and recurrent pleural effusions without ascites. We hope to support the conclusion that a patient with recurrent pleural effusions, without ascites, does not exclude gastrointestinal involvement in its etiology.

## INTRODUCTION

Hepatic hydrothorax is defined as a significant pleural effusion (usually >500 ml) that develops in a patient with cirrhosis of the liver and portal hypertension, in the absence of cardiopulmonary disease. It is right sided in most cases, develops in 6–10% of patients with end-stage liver disease, and is commonly seen in conjunction with ascites. Very rarely, however, it can present in the absence of ascites[1,2,3,4,5,6,7,8,9]. We report a case of recurrent unilateral pleural effusion in a patient with no clinical or radiologic evidence of ascites. A brief review of this rare presentation of hepatic hydrothorax is included.

## CASE PRESENTATION

A 50-year-old female was admitted with a 1-week history of worsening dyspnea and right-sided pleuritic chest pain. She denied any fever, hemoptysis, or a similar previous episode. Past medical history is significant for hypertension and hepatitis C. Her hepatitis C was contracted through IV drug abuse and diagnosed 2 years ago after a routine workup. She refused treatment for hepatitis C and never followed up with any physician. The patient continues to be noncompliant with treatment for hepatitis C. She currently denies drug use and only drinks socially.

The patient's last hospital admission was 8 months ago at another institution for gastroenteritis and Gastroenterology was consulted to manage her hepatitis C. She followed up with a gastroenterologist and had a liver biopsy done 2 months ago, which showed early cirrhosis. The Metavir score was F (fibrosis) = stage 3 and A (activity) = grade 2; focal periportal fibrosis with bridging between portal structures without nodule formation and moderate activity/inflammation involving periportal and lobular necrosis.

Physical examination showed a blood pressure of 129/62 mmHg, pulse of 83 bpm, respiratory rate of 22/mt, and temperature of 97.4°F. Sclerae were anicteric and there was no jugular venous distension. Chest examination revealed decreased breath sounds with percussion dullness and decreased vocal fremitus over the lower three-fourths of the right hemithorax. Cardiovascular and abdominal examinations were unremarkable. There was no pedal edema or clubbing.

The initial chemistry revealed albumin of 3.5 g/dl, total protein of 7.7 g/dl, creatinine of 0.9 mg/dl, LDH of 211 IU/l, AST of 134 IU/l, ALT of 99 IU/l, and alkaline phosphatase of 111 IU/l. Hemogram was unremarkable with platelets of 212/$mm^3$ and INR of 1.08. Renal function was within normal limits with a creatinine of 0.9 mg/dl and BUN of 7 mg/dl. Hepatitis C antibody was positive and genotyping revealed genotype 2b. Chest roentgenogram showed massive right-sided pleural effusion (Figs. 1 and 2). CT scan (Fig. 3) as well as ultrasonogram of the abdomen showed fatty liver, but no ascites. The patient underwent right thoracentesis with a return of 2 l of straw-colored fluid, which on analysis revealed PH of 7.65, LDH of 60 IU/l, protein of <1 g/dl, glucose of 104 mg/dl, and WBC of 78/$mm^3$. Bacterial and fungal cultures of the pleural fluid were negative, as was the cytology. Although her blood work was not consistent with cirrhosis, she had an upper endoscopy revealing gastroesophageal varices, which was consistent with portal hypertension and cirrhosis. She was started on salt-free diet, diuretics, and beta blockers, and was discharged with a 2 week follow-up appointment. She followed up in the family care clinic 2 weeks later and had similar complaints of dyspnea and pleuritic chest pain. Physical exam showed dullness to percussion on the right side of the chest. No shifting dullness or fluid wave was appreciated. CXR again revealed a moderate-size effusion. Patient was admitted for thoracentesis and 1.8 l of fluid was again removed. Chemistries were very similar to last admission and showed transudative effusion. Two days later, a repeat CXR showed recurrent effusion. In order to determine the etiology of the recurrent effusions, it was necessary to rule out any cardiac, renal, or GI causes of effusion. A renal workup showed a normal BUN and creatinine. A cardiology workup revealed normal left ventricular wall motion and contractility on echocardiogram. Abdominal ultrasound showed splenomegaly, which was confirmed by CT. Among the etiologies of recurrent effusions, only the GI workup (portal hypertension, esophageal varices, fatty liver, splenomegaly, and early cirrhosis) had a positive correlation with the patient's presentation.

Because of her recurrent effusions and coinciding dyspnea and chest pain, she underwent video-assisted thoracoscopic surgery (VATS) with talc pleurodesis of the right chest. As per the surgeon, there were no visible diaphragmatic defects. The patient tolerated the procedure well and had a chest tube placed to remove any excess pleural fluid. The patient continued to drain about 1 l of fluid from the chest tube for about 3 days. The drainage eventually slowed and stopped in the next 4 days. Three days following the procedure, the patient denied any more dyspnea or chest pain, but continued to drain fluid into her chest tube. Ten days following the VATS, the chest tube discontinued draining fluid and the patient was discharged with significant clinical improvement.

## DISCUSSION

Isolated hepatic hydrothorax in the absence of ascites is rare and provides diagnostic confusion. Hepatic hydrothorax occurs as a result of ascitic fluid leaking from the peritoneal cavity into the pleural space through defects in the tendinous portion of the diaphragm (mostly <1 cm in diameter). Diaphragmatic defects are created from pleuroperitoneal blebs that rupture and when there is an increase in intra-abdominal pressure from coughing, straining, or ascites[1,2,3,4,5,6,7]. Due to the negative intrathoracic pressure that occurs during breathing, a unidirectional flow of ascitic fluid to the pleural space occurs through

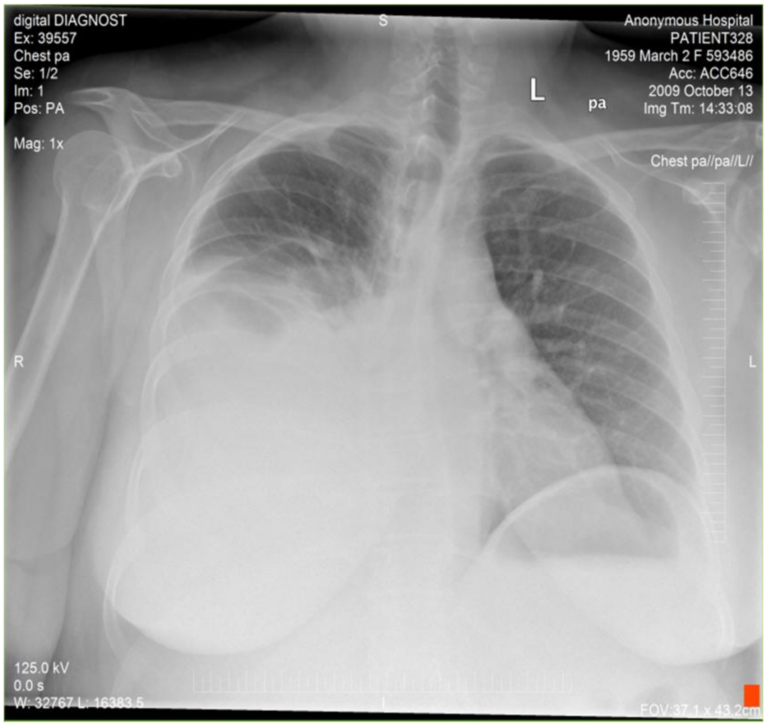

**FIGURE 1.** Posterior to anterior (PA) CXR showing right-sided pleural effusion.

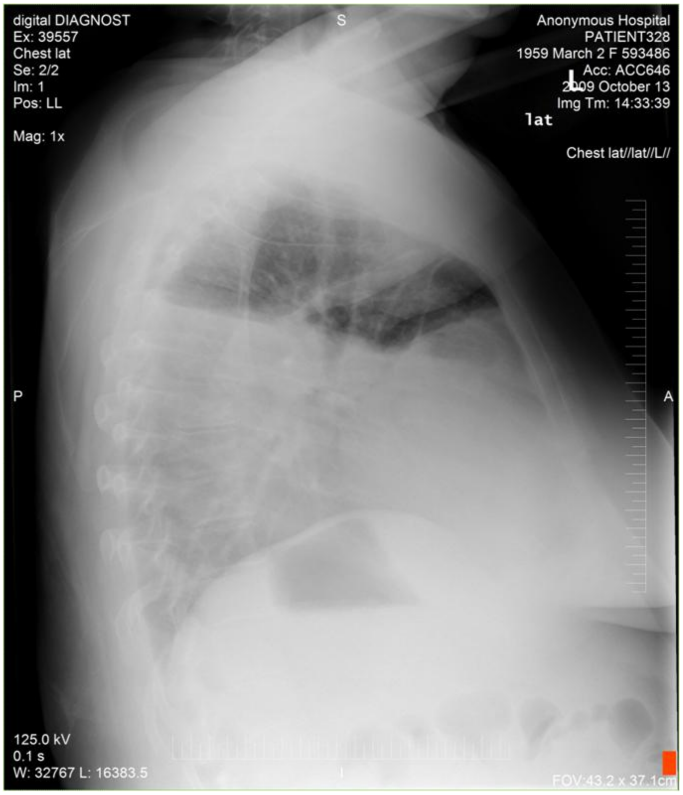

**FIGURE 2.** Lateral CXR showing right-sided pleural effusion.

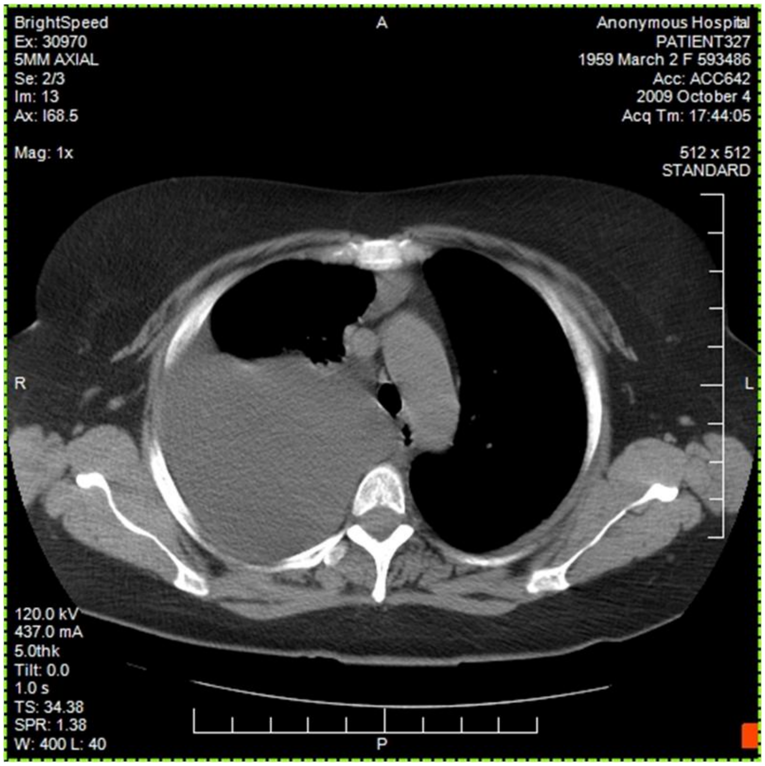

**FIGURE 3**. Axial thoracic CT showing right-sided pleural effusion.

these diaphragmatic defects[1,2,10]. This same mechanism can also be applied to hepatic hydrothorax without ascites. The difference between hepatic hydrothorax without ascites and that with ascites is that the diaphragmatic defects are larger and more persistent in these cases. The rate of the formation of ascitic fluid does not exceed the capacity of the ascitic fluid to be absorbed and drained from the peritoneal cavity by way of a pressure gradient[3,10].

In order to diagnose hepatic hydrothorax, one must first exclude any cardiopulmonary or renal dysfunction. Also required is the presence of transudative pleural fluid and the demonstration of diaphragmatic defects when possible[1,2,3,4,5,6,7,8,9,10]. However, sometimes the diaphragmatic defects are too small to be visualized on gross examination. The intraperitoneal injection of a radioisotope (Tc99m or methylene blue) can confirm the movement of ascitic fluid into the pleural cavity through the diaphragmatic defects. In hepatic hydrothorax, the 99mTc-sulfur colloid does not localize in the pleural cavity in patients with pleural effusion secondary to cardiopulmonary disease[6]. Intraperitoneal spraying of indiocyanine green (ICG) is a newer proposed method of diagnosing hepatic hydrothorax. The technique described is used only if the patient is continually draining fluid from the pleural effusion because the fluid from the effusion needs to be analyzed immediately after the spraying of the ICG. The concentration of ICG collected from the pleural space after being injected into the peritoneal cavity should be significantly diluted by the pleural effusion to show proof of a peritone-pleural flow[9].

Initial management of hepatic hydrothorax includes sodium-restricted diet and diuretics with consideration for orthotopic liver transplantation. If medical treatment and therapeutic thoracentesis are needed repeatedly, other options are available. Transjugular intrahepatic portosystemic shunt (TIPS) or VATS repair of the diaphragmatic defects (with or without pleurodesis) are effective strategies in those who are not transplant candidates or those awaiting organ availability[1,2,3,4,5,6,7,8,9].

## CONCLUSION

Although hepatic hydrothorax usually presents with ascites, there are rare cases when it does not. We present a case of hepatic hydrothorax in a patient with hepatitis C and liver cirrhosis that has no clinical evidence of ascites. VATS and talc pleurodesis were performed and the patient's recurrent effusions resolved.
